# Mariage non consommé et vaginisme: à propos de trois cas Clinique

**DOI:** 10.11604/pamj.2017.27.60.6694

**Published:** 2017-05-29

**Authors:** Rabie Karrouri

**Affiliations:** 1 Service de Psychiatrie, Hôpital Militaire Moulay Ismail, Meknès 50000, Maroc

**Keywords:** Vaginisme, mariage non consommé, pénétration, Maroc, couple, Vaginismus, non-consummation of marriage, penetration, Morocco, couple

## Abstract

Le vaginisme est un problème de couple. Il est source de mariages non consommés, d’infertilité et d’altération de la qualité de la relation sexuelle du couple. Par trois cas cliniques illustratifs rapportés de notre pratique clinique quotidienne et suivis sur deux années en consultation du service de psychiatrie de l’Hôpital Militaire Moulay Ismail de Meknès, nous essayons de clarifier les motifs de rencontre pour vaginisme, ses aspects cliniques et relationnels et ses particularités culturelles.

## Abstract (en)

Vaginismus is a relationship issue. It is a cause of non-consummation of marriage, infertility and alteration in sexual relationship quality. We present three case reports from our daily clinical practice in order to highlight the possible causes of vaginismus, its clinical and relational characteristics and its cultural particularities. Patients were treated in the Department of Psychiatry at the Military Hospital Moulay Ismail, Meknes, over an observation period of two years.

## Introduction

Le vaginisme, qui est une impossibilité d’avoir des rapports sexuels avec pénétration vaginale [1] est définit par une contracture involontaire du tiers externe du vagin empêchant toute pénétration [2]. Il est souvent associé à une contracture plus globale de toute la région, abdominal, fessier, adducteurs des cuisses, empêchant ainsi tout approchement du partenaire. Il faut le distingué de la dyspareunie qui est une douleur vaginale persistante ou récidivante associé à une pénétration [2] et ce bien que la distinction est parfois difficile surtout avec la dyspareunie superficielle, d’autant plus que douleur et contractures musculaires peuvent aller de pair [2]. Malgré le climat apparent de ‘‘libération sexuelle’’, consulter pour parler des difficultés sexuelles reste encore un tabou dans pratiquement toutes les sociétés, avec des variations légères d’une société à l’autre. Le vaginisme est vu chez environ 0.5% des femmes [1] et 12.5% des mariages non consommés [3]. Au Maroc nous n’avons pas de données spécifiques sur la prévalence du vaginisme, mais n’en reste pas moins un des dysfonctions sexuelles les plus difficiles à prendre en charge et à traiter efficacement [3]. Par trois cas cliniques, suivis en ambulatoire dans le service de psychiatrie de l’Hôpital Militaire Moulay Ismail de Meknès, nous allons essayer de clarifier certains aspects cliniques et culturelles du vaginisme chez la femme marocaine.

## Patient et observation

### Cas clinique 1

Patiente âgée de 20 ans, femme au foyer, nous a été adressé 01 mois après la naissance de sa première fille par le médecin du centre de santé où la patiente suivait sa grossesse, et ce pour un mariage non consommé de 09 mois. La patiente est décrite par son médecin de « non coopérante » aux examens gynécologiques de surveillance de la grossesse et à l’accouchement. Accompagnée par son mari, la patiente rapporte une angoisse importante face à la pénétration vaginale. Durant 09 mois d’un mariage arrangé, mais avec le consentement des deux partenaires, la patiente n’a eu que des rapports sexuels superficiels alors que toute pénétration était impossible selon son mari, « c’est comme percuter sur un mur » dit-t-il. L’accouchement était par césarienne vu que la patiente refusait tout examen gynécologique aussi bien avant le travail qu’au cours de celui-ci. L’entretien n’a pas pu mettre en évidence une notion d’agression ou d’abus sexuel dans ses antécédents mais plutôt une grande influence des dires de ses cousines mariées qui décrivaient la douleur de défloration d’horrible et insupportable. Education sexuelle avec une psychothérapie de soutien a permis de réduire sensiblement l’anxiété de la patiente pendant le rapport sexuel et à l’examen gynécologique mais n’a pas permis pour autant la pénétration.

### Cas clinique 2

Patiente âgée de 23 ans, agent comptable, qui nous consulte pour un tableau clinique d’une dépression majeur (tristesse, anhédonie, irritabilité) avec un trouble panique (palpitation, sensation d’étouffement et de mort imminente, tremblement) avec un sommeil perturbé. Une psychothérapie de soutien avec un traitement antidépresseur lui a était procurer et ayant permis d’obtenir une amélioration partielle au bout de 02 mois de traitement. L’entretient poussé abordant surtout la question d’infertilité a permis à la patiente de rapporter une notion de mariage non consommé de 03 ans. Malgré le désir sexuel qu’elle ressent et sa volonté de consommer son mariage pour pouvoir procréer, elle craint toute pénétration par peur de saigner abondamment sans pouvoir l’arrêter. L’acte sexuel est bien préparé, habits et environnement, mais reste toujours bloqué aux préliminaires et toute tentative de son mari pour l’approcher en guise de pénétration est rapidement vouée à l’échec par une fermeture serrée de ses cuisses tout en poussant activement son partenaire. Toutefois elle arrive presque régulièrement à avoir un orgasme satisfaisant. Mariée après « une longue histoire d’amour », sans notion d’attouchement sexuel avant le mariage, interrompu formellement par la patiente, « je ne le laisse même pas y penser » disait-elle. Son mari plus âgé qu’elle de cinq ans est décrit de « fils à maman ». Ambivalente sur sa plainte, elle se focalise sur la pression exercée par son entourage familial pour le retard de grossesse et rejette la responsabilité de son trouble sur son mari qui le décrit de « n’est pas courageux » et « peureux » lors des rapports sexuels. Le couple a bénéficié d’une psychothérapie de soutient avec des exercices de relaxations ayant permis de diminuer l’anxiété des deux partenaires et l’opposition de la patiente et ainsi rendre un rapport superficiel possible.

### Cas cliniques 3

Patiente âgée de 19 ans, élève du lycée, fiancée à son cousin maternel depuis 05 mois, le mariage prévu dans 04 mois. Elle Consulte pour avoir un conseil et une prise en charge de ses craintes concernant la nuit de noce, surtout qu’elle se voit obliger de prouver la consommation de son mariage la fin de cette nuit. La patiente éprouve une crainte et une peur importante concernant la nuit de noce et surtout la notion de pénétration vaginale, elle rapporte une peur panique d’éclater par pénétration voir de mourir suite à une hémorragie secondaire à la défloration. Décrivant son futur mari d’être gentil, compréhensif et tendre, elle se plaint surtout des comtes de ses tantes, cousines et amies sur la douleur produite par le rapport sexuel. Elle ne rapporte aucun antécédent d’agression ou d’abus sexuel. La patiente a bénéficié d’une psychothérapie de soutien apportant explication sur la physiologie et anatomie du rapport sexuel. Perdue de vue après cette consultation, le devenir du mariage n’a pas pu être connu.

## Discussion

Consulter un psychiatre pour un problème de vaginisme parait plus facile de nos jours. La consultation était motivée par deux raison essentielle implicite ; la recherche de satisfaction sexuelle, source de consultation précoce et explicite ; la recherche de procréation, source d’un retard de consultation. Le délai de consultation chez nos patientes est relativement précoce contrairement à ce qui est relaté par plusieurs études qui rapportent un moyen de plusieurs années [4,5]. Ceci est peut-être dû dans notre culture à la préservation des relations familiales fortes voir fusionnelles, permettant à l’entourage de se demander sur le retard de la procréation, sans considérer l’intimité de ce sujet, sinon à la diffusion de l’information quant à l’importance de la qualité de la relation sexuelle dans la vie de couple.

Le vaginisme est un problème de couple voir de famille, la demande d’aide peut être faite par la femme (cas clinique 2), son époux (cas clinique 1&2), voir sous pression de la famille qui se demande sur le retard de procréation (cas clinique 2) sinon de la virginité de la femme après consommation du mariage (cas clinique 3). Le vaginisme n’est pas toujours source d’infertilité pourtant il faut le chercher systématiquement devant tout retard de procréation [2,5].

Si ces femmes expriment le plus souvent une peur de la pénétration voire un vaginisme, leur partenaire adopte plutôt une attitude passive, non intrusive, voire bienveillante [6] recherchant progressivement d’autres moyens de satisfaction sexuelle. Ceci a pour conséquence une pérennisation du trouble mais paradoxalement, la préservation de la vie du couple évoluant pour certains auteurs à une relation proche de la fraternité [6]. Le divorce dans les couples qui se plaignent de vaginisme est ainsi plus rare que dans les couples dits « normaux » [7], en dehors des cas de mariage forcé, les patientes prennent généralement leur temps avant de se faire choisir le mari « convenable » à leurs « craintes » (cas clinique 2 et 3).

On n’a pas pu mettre en évidence d’antécédents de traumatisme à l’enfance notamment un abus sexuel, décrit par certains auteurs [6], ni de troubles psychiatriques caractérisés, mais la plupart de nos patientes portent des symptômes anxieux manifeste souvent plus accentués chez leur mari. Classiquement une éducation répressive était longtemps mise en cause [6], mais on voit le vaginisme surgir aujourd’hui dans un contexte permissif où on peut même voir des consultantes pour une prise en charge précoce voir préventif (cas clinique 3) pour un trouble qui ne peut être classé à ce stade que de probable et non confirmer. Le vaginisme nous paraît plus secondaire à l’influence de l’entourage proche des patientes et surtout par des femmes se déclarant d’être « expérimentées » et qui favorise le développement de cette angoisse concernant le rapport sexuel et plus particulièrement la pénétration.

## Conclusion

Si le vaginisme est une dysfonction sexuelle féminine, en pratique quotidienne c’est une dysfonction du couple. Consulter un psychiatre pour un problème de vaginisme est plus facile de nos jours. Le développement de ce trouble semble plus en rapport avec l’influence de l’entourage des patientes. Toutefois seule une étude plus étendue sur le temps et concernant un échantillon plus vaste peuvent confirmer ou rejeter de telle supposition. L’intérêt de l’éducation sexuelle orientée vers les futurs mariés et respectant les convictions culturelle et religieuse des sociétés peut jouer un rôle important dans la prévention du développement de tel trouble voir faciliter une consultation précoce avant sa pérennisation et l’altération de la dynamique du couple.

## Conflits d’intérêts

Les auteurs ne déclarent aucun conflit d’intérêts.
